# The hepatitis E virus ORF1 hypervariable region confers partial cyclophilin dependency

**DOI:** 10.1099/jgv.0.001919

**Published:** 2023-11-09

**Authors:** Frazer J. T. Buchanan, Shucheng Chen, Mark Harris, Morgan R. Herod

**Affiliations:** ^1^​ School of Molecular and Cellular Biology, Faculty of Biological Sciences and Astbury Centre for Structural Molecular Biology, University of Leeds, Leeds, LS2 9JT, UK; ^2^​ Department of Paediatrics, Harvard Medical School, Boston, Massachusetts, USA

**Keywords:** cyclosporine, HEV, replication complex, replicon

## Abstract

Hepatitis E virus (HEV) is an emerging pathogen responsible for more than 20 million cases of acute hepatitis globally per annum. Healthy individuals typically have a self-limiting infection, but mortality rates in some populations such as pregnant women can reach 30 %. A detailed understanding of the virus lifecycle is lacking, mainly due to limitations in experimental systems. In this regard, the cyclophilins are an important family of proteins that have peptidyl-prolyl isomerase activity and play roles in the replication of a number of positive-sense RNA viruses, including hepatotropic viruses such as hepatitis C virus (HCV). Cyclophilins A and B (CypA/B) are the two most abundant Cyps in hepatocytes and are therefore potential targets for pan-viral therapeutics. Here, we investigated the importance of CypA and CypB for HEV genome replication using sub-genomic replicons. Using a combination of pharmacological inhibition by cyclosporine A (CsA), and silencing by small hairpin RNA we find that CypA and CypB are not essential for HEV replication. However, we find that silencing of CypB reduces replication of some HEV isolates in some cells. Furthermore, sensitivity to Cyp silencing appears to be partly conferred by the sequence within the hypervariable region of the viral polyprotein. These data suggest HEV is atypical in its requirements for cyclophilin for viral genome replication and that this phenomenon could be genotype- and sequence-specific.

## Introduction

Hepatitis E virus (HEV) is one of the leading aetiological agents of acute hepatitis and is responsible for more than 20 million cases annually. The virus is a member of the genus *Paslahepevirus* within the family *Hepeviridae*. The genus has two species, *Paslahepevirus alci* which includes HEV variants from moose, and *Paslahepevirus balayani* which can infect a wide range of animals, including humans. *P. balayani* is currently sub-classified into eight genotypes (termed HEV-1 to HEV-8) [1–5]. HEV-1 and HEV-2 appear to be obligate human pathogens that are transmitted between humans by the faecal–oral route, with the potential to cause large outbreaks [5, 6]. HEV-3 and HEV-4 have been isolated in several animal species including humans and have the potential for zoonotic transmission [7, 8]. Infection in healthy individuals usually leads to acute hepatitis which has a low rate of mortality. However, infection during pregnancy is of particular concern as mortality rates have been reported to be up to 30 % [9]. This higher risk of mortality has also been observed in immunocompromised individuals. No specific treatment is available for HEV-infected individuals, with antivirals such as ribavirin used in combination with supportive care [10].

**Fig. 1.**
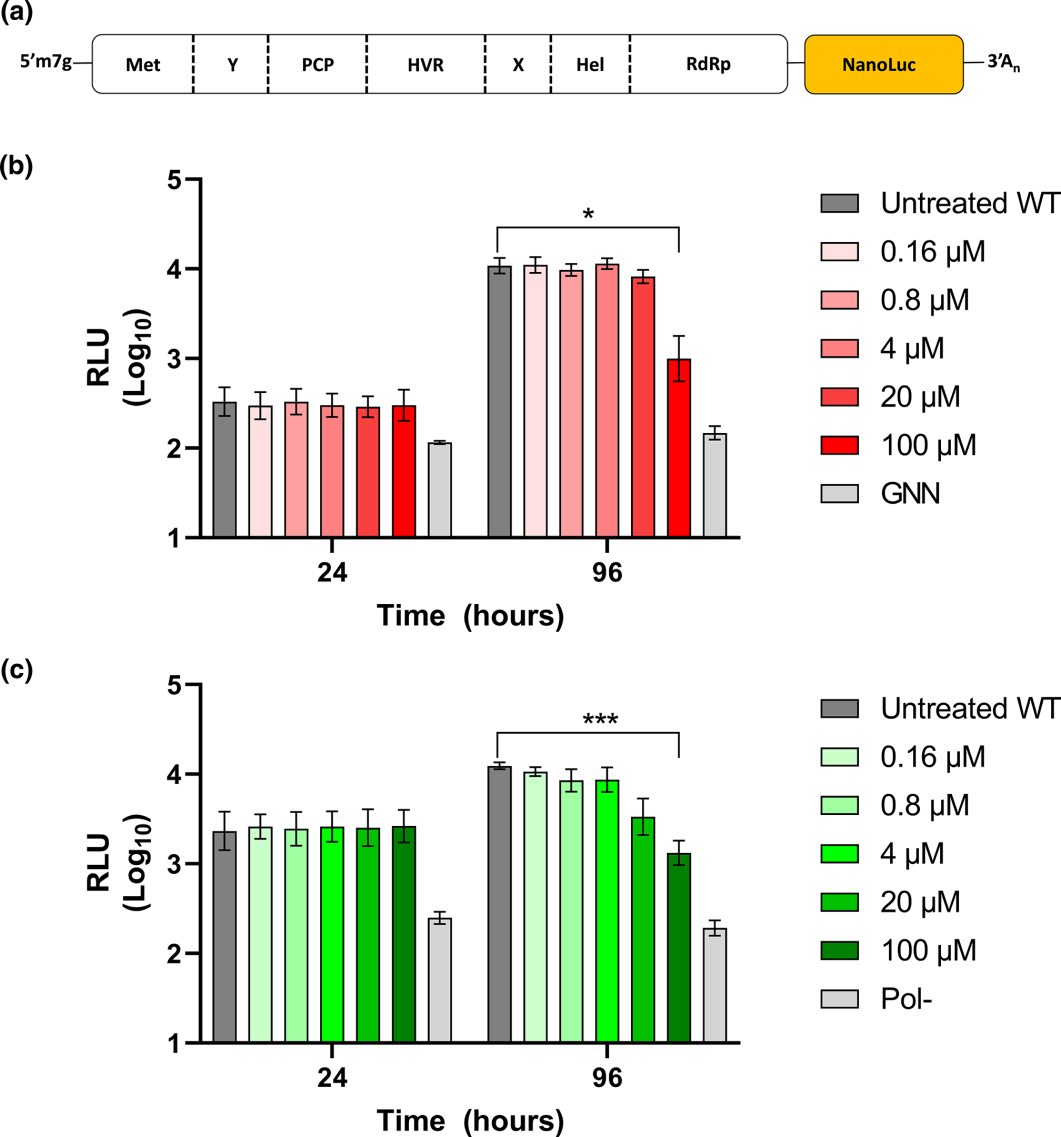
CsA dose response in HEV transfected hepatocytes. (**a**) Schematic of HEV replicon genome showing ORF1 and ORF2/3 are replaced with a nano-luciferase reporter (nLuc). ORF1 is reported to contain a methyltransferase (Met), Y domain (Y), putative cysteine protease (PCP), hypervariable region (HVR), macro domain (X), helicase domain (Hel) and RNA-dependent RNA polymerase (RdRp). ORF2 and ORF3 are both produced from a viral sub-genomic RNA. (**b**) Huh7 cells were electroporated with wild-type (WT) SK-E2-nLuc or SK-E2-nLuc-GNN (GNN) SGR RNA prior to addition of CsA at varying concentrations (0–100 µM) 24 h post-electroporation. Cells were harvested at 24 h intervals for 120 h and luciferase activity was determined. Data are presented as mean luciferase activity as relative light units (RLU) (*n*=3, ±sem). (**c**) Huh7 cells were electroporated with either WT 83-2-nLuc replicon or 83-2-nLuc-Pol- (Pol-) replication defective control SGR RNA. Cells were harvested at 24 h intervals for 120 h and luciferase activity was determined. Data are presented as mean luciferase activity as RLU (*n*=3, ±sem, ***P<0.001, **P<0.01; 100 µM compared to untreated WT).

HEV is a single-stranded positive-sense RNA virus with a genome length of approximately 7.2 kb. The genome contains three ORFs. ORF1 is translated to produce the viral polyprotein that contains the protein domains required for viral RNA replication. The second and third ORFs, ORF2 and ORF3, are translated into the viral capsid protein and a small membrane protein involved in virus release, respectively [11]. A fourth ORF, ORF4, has also been identified but only in HEV-1 [12]. The ORF1 polyprotein, also known as pORF1, is necessary and sufficient for viral genome replication. Through sequence homology to related virus families pORF1 has been predicted to contain at least six distinct protein domains (Fig. 1a) [13]. At the N-terminus of the polyprotein is a methyltransferase (MeT) domain which has been biochemically characterized and suggested to interact with the adjacent Y domain or ‘iceberg region’ [14, 15]. MeT-Y is followed by the putative cysteine protease (PCP) domain. Spanning the centre of the polyprotein is a stretch of high sequence diversity termed the hypervariable region (HVR) with no known function, which is followed by the X region or macrodomain that can bind and hydrolyse ADP-ribose and is required for genome replication. At the C-terminus of the polyprotein are domains with helicase (Hel) and RNA-dependent RNA-polymerase (RdRp) activity [16–20].

HEV is a hepatotropic virus with hepatocytes being the primary site of infection and pathology. Small molecules that inhibit the replication of hepatotropic viruses such as hepatitis C virus (HCV) have shown promise as therapies as well as tools for understanding fundamental virus biology. In this regard, the cyclophilins (Cyps) are a family of peptidyl prolyl isomerases that function in a number of cellular processes such as protein folding, trafficking and innate immune signalling, and have been identified as proteins that are co-opted by viruses to promote their replication. CypA is the predominant human Cyp which has been documented to be important for the replication of a number of viruses including severe acute respiratory syndrome coronavirus 2 (SARS-CoV-2), human immunodeficiency virus (HIV) and HCV [21, 22]. In the case of HCV, CypA is bound by the viral non-structural protein NS5A and this has been reported to inhibit protein kinase R (PKR), and aid evasion of the innate immune system [21, 22]. Additionally, CypB has also been reported to be important for HCV replication [21], and cyclosporine A was suggested as a potential treatment for HCV [23].

The literature regarding the role of the Cyps in HEV infection is currently limited. Wu *et al*. [24] reported that CypA disruption did not impact the replication of HEV primary isolates in cell culture. Contrary to this, Wang *et al*. [25] reported that inhibition of CypA with cyclosporine A (CsA) promotes HEV replication. Given the importance of CypA and CypB in the replication of other chronic hepatotropic viruses, they represent possible pan-therapeutic targets. We therefore decided to investigate thoroughly a potential role for these proteins in HEV replication. Using sub-genomic replicons (SGRs) of HEV, we compared the effect of pharmacological and genetic silencing of CypA and CypB on viral genome replication. Our data suggest that CypA or CypB is not essential for HEV replication but can increase replication efficiency in some cell types. Furthermore, using chimeric SGRs the sequence of the viral HVR is implicated as an important determinant for replication fitness and Cyp dependency.

## Methods

### Cell lines and plasmids

Huh7, Huh7.5 and HEK293T cells were maintained in Dulbecco’s modified Eagle’s medium with glutamine (Sigma-Aldrich) supplemented with 10 % FCS, 1× non-essential amino acids (Gibco), 50 U ml^−1^ penicillin and 50 µg ml^−1^ streptomycin (Sigma-Aldrich).

A plasmid carrying the wild-type HEV-1 (Sar55 strain; GenBank accession no. AF444002) SGR expressing GFP, pSK-E2-GFP, was a kind gift from Dr Patrizia Farci and has been described previously [26]. This plasmid was modified to replace the GFP ORF with nano-luciferase as previously described [26] to produce pSK-E2-nLuc. Mutations within these plasmids were performed by standard two-step overlapping PCR mutagenesis. A negative control SGR pSK-E2-nLuc-GNN was generated containing a double point mutation in the RdRp active site GDD motif (GNN) and has been previously described [27]. A plasmid containing the wild-type HEV-3 SGR expressing nano-luciferase, pUC-HEV-83-2, was a kind gift from Dr Koji Ishii and has been described previously [28, 29]. A negative control pUC-HEV-83-2-pol- SGR was generated by engineering a frame-shift in the RdRp coding sequence by digestion with *Kpn*I and Mung Bean nuclease (NEB) treatment followed by re-ligation. Chimeric pUC-HEV-83-2 replicons containing Kernow-C1/P6 sequences were generated by standard two-step overlapping mutagenesis.

### Generation of silenced cell lines

HEK293T cells in 10 cm dishes were transfected with 1 µg packaging plasmid p8.91, 1 µg envelope plasmid pMDG encoding VSV-G protein and 1.5 µg transfer plasmid pHIV-SIREN encoding short hairpin RNA (shRNA) to CypA or CypB as described [19]. Lentivirus supernatants were collected at 48 h and filtered through a 0.45 µm syringe. Huh7 or Huh7.5 cells were seeded into six-well plates at a density of 2.5×10^5^ cells per well and transduced with 1 ml per well lentivirus and 8 µg ml^−1^ polybrene for 24 h. Transduced cells were selected using 2.5 µg ml^−1^ puromycin at 72 h post-transduction.

### *In vitro* transcription

pSK-E2-nLuc and pUC-HEV-83-2 replicon plasmids were linearized with *Bgl*II or *Hind*III respectively before being used to generate T7 *in vitro* transcribed RNA using the HiScribe T7 ARCA mRNA kit with tailing following the manufacturer’s instructions (Promega). RNA was purified using an RNA clean and concentrate kit (Zymo Research) and the quality was checked using a MOPS/formaldehyde agarose gel electrophoresis.

### Replication assays

Replicon experiments were conducted as previously described [27]. Briefly, Huh7 or Huh7.5 cells were detached by trypsin, washed twice in ice-cold DEPC-treated PBS and re-suspended at 1×10^7^ cells ml^−1^ in DEPC-treated PBS. Subsequently, 400 µl of cells was mixed with 2 µg of RNA transcript, transferred to a 4 mm gap electroporation cuvette (SLS) and pulsed at 260 V, 25 ms pulse length, in a Bio-Rad Gene Pulser (Bio-Rad) on the square wave setting. Electroporated cells were recovered into 4 ml media and seeded into replicate six-well tissue culture plates, and replication was measured at 24 h intervals using the Nano-Glo luciferase assay system (Promega). For CsA treatment the electroporated cells were seeded into replicates of 24-well plates, allowed them to adhere before the media were replaced with fresh media containing CsA (all Sigma-Aldrich), at the indicated concentration. Statistical analysis was conducted using unpaired t-tests using GraphPad PRISM software (*P<0.05, **P<0.01 and ***P<0.001).

### MTS assay

The cell viability experiments were conducted by seeding cells into 96-well plates, allowing adherence for 24 h before addition of a serial dilution of inhibitor and measurement of cell viability 72 h later using the CellTiter AQueous One solution (Promega), following the manufacturer’s instructions. Briefly, 20 µl of reagent was added to each well before samples and appropriate media-only blanks were incubated for 45–60 min at 37 °C. Absorbance at 490 nm was measured on an Infinite F50 (Tecan).

### Western blotting

Cell lysates were centrifuged for 20 min at 17 000 *g*, supernatant removed to a separate tube and mixed with an equal volume of 2× Laemmli buffer (Sigma-Aldrich). Samples were heated for 5 min at 100 °C and separated on a 10 % SDS polyacrylamide gel. Proteins were transferred onto Immobilon transfer membrane (Merck) using a BioRad Trans-Blot turbo transfer system. Membranes were blocked in 10 % milk in Tris-buffered saline solution containing 0.1 % Tween (Fisher). Membranes were then incubated overnight at 4 °C with rabbit anti-CypA (1 : 1000) (Enzo) or anti-CypB (1 : 2000) (Abcam) antibody. Membranes were washed three times prior to 1 h of incubation with anti-rabbit HRP-conjugated secondary antibody. Membranes were washed three times and incubated in ECL reagent (Thermo scientific) before exposure to CL-Xposure film (Thermo scientific), and developed by XOgraph (Fuji). Quantification of Western blots was performed by densitometry using ImageJ software.

## Results

### Pharmacological inhibition of cyclophilin does not impact HEV genome replication

Previous work has established that functional CypA is necessary to support the replication of several positive-sense RNA viruses, such as HCV [21, 22, 24]. However, the role for Cyps in the replication of HEV remains disputed, in part due to the difficulty in investigating separate parts of the viral replication cycle in isolation. To elucidate the effects of Cyps on HEV genome replication we employed an HEV SGR, a self-replicating RNA in which a portion of the coding sequence for the viral structural proteins is replaced by a nano-luciferase (nLuc) reporter gene (Fig. 1a). Measurement of nLuc activity allows for an indirect measure of viral genome replication in the absence of virus entry or assembly. CsA is a potent inhibitor of both CypA and CypB. It is a cyclic molecule derived from the fungus *Tolypocladium inflatum* and complexes with Cyp to block peptidyl prolyl isomerization as well as preventing interactions with other cellular proteins [30–32]. We therefore decided to start by investigating the sensitivity of HEV replication to CsA.

Two human hepatocellular carcinoma lines that support HEV replication (Huh7 and the derivative cell line Huh7.5, which contain a RIG-I mutation and support enhanced replication of viruses such as HCV [33, 34]) were transfected with an HEV-1 SGR RNA (SK-E2-nLuc), derived from the Sar55 infectious clone sequence [26] (Fig. 1a). As a negative control, cells were also transfected with a replication-defective SGR (SK-E2-nLuc-GNN), which contained two inactivating mutations in the active site of the viral RNA polymerase. CsA was added to the growth medium 24 h after electroporation at varying concentrations (0–100 µM), and replication was assayed daily for 120 h post-electroporation (Figs 1b and S1).

For the wild-type (WT) untreated SGR, nLuc activity increased approximately 100-fold over the duration of the experiment, whereas the replication defective replicon (GNN) only exhibited background levels of nLuc activity at every time point. In comparison to the untreated WT SGR there was no marked difference in nLuc activity upon treatment of CsA up to a concentration of 20 µM. In contrast, there was approximately an 11-fold decrease in nLuc activity in cells treated with 100 µM CsA compared to untreated cells by 96 h post-electroporation. The pattern of results remained consistent in Huh7.5 cells when compared to Huh7 cells, suggesting both cell lines are able to support replication to a similar level (Fig. S1, available in the online version of this article). These data suggest only the highest (and cytotoxic) concentration of CsA used (100 µM) reduced luciferase activity.

The effect of the Cyp proteins on HEV replication has yielded conflicting results, which could potentially be the consequence of variances in the viral genotypes investigated. It was therefore considered important to extend our investigations to include another genotype of human importance; we chose to focus on HEV-3 as infectious clones and SGR are available for this genotype. Therefore, Huh7 cells were transfected with an HEV-3 SGR RNA (83-2-nLuc), which is derived from the G3-HEV83-2-27 infectious clone sequence. As before cells were also transfected with an equivalent replication defective control SGR (83-2-nLuc-Pol-). CsA was added to the growth medium 24 h after electroporation at varying concentrations (0–100 µM), and replication was assayed daily for 120 h post-electroporation (Figs 1 and S1).

In comparison to the untreated WT 83-2-nLuc SGR there was no significant difference in nLuc activity upon treatment of CsA up to a concentration of 20 µM. However, as with the WT HEV-1 SK-E2-nLuc SGR, there was approximately a 10-fold decrease in nLuc activity in cells treated with 100 µM of CsA compared to untreated cells by 96 h post-electroporation.

Both the data with the HEV-1 SK-E2-nLuc and HEV-3 83-2-nLuc SGRs suggest that reduced replication as only observed at CsA concentrations above 20 µM, which have been reported to be cytotoxic [35, 36]. To quantify any difference between cytotoxicity and inhibition of replication we conducted comparative cytotoxicity experiments. Huh7 and Huh7.5 cells were treated with a serial dilution of CsA and cytotoxicity was evaluated by an MTS assay 96 h post-treatment (Fig. S1). Cytotoxicity was similar in both Huh7 and Huh7.5 cells with >75 % viability at concentrations of ≤20 µM. However, at 100 µM Huh7 and Huh7.5 cells showed average cell viability of ~56 %, which is similar to the reduction in nLuc activity observed (Fig. 1). Taken together these data suggest that CsA treatment at 24 h post-electroporation does not reduce HEV replication at sub-cytotoxic concentrations. To investigate if pre- or post-treatment of cells with CsA could inhibit replication, we next conducted a time-of-addition assays with both SK-E2-nLuc and 83-2-nLuc SGR RNAs. Huh7 cells were pre-treated with 20 µM CsA 24 h prior to, immediately after or 8 h after electroporation with either SK-E2-nLuc or 83-2-nLuc SGR RNAs and replication assayed daily for 120 h post-electroporation (Fig. S1). With either SK-E2-nLuc or 83-2-nLuc SGR RNAs there was no significant difference in nLuc activity at any timepoints assayed. Thus it appeared that pre- or post-treatment with CsA did not inhibit HEV replication at sub-cytotoxic concentrations. Taken together these data suggest that CsA treatment does not reduce HEV replication at sub-cytotoxic concentrations and we conclude from these data that pharmacological inhibition of Cyps does not affect HEV replication.

### CypA is not essential for HEV replication

**Fig. 2.**
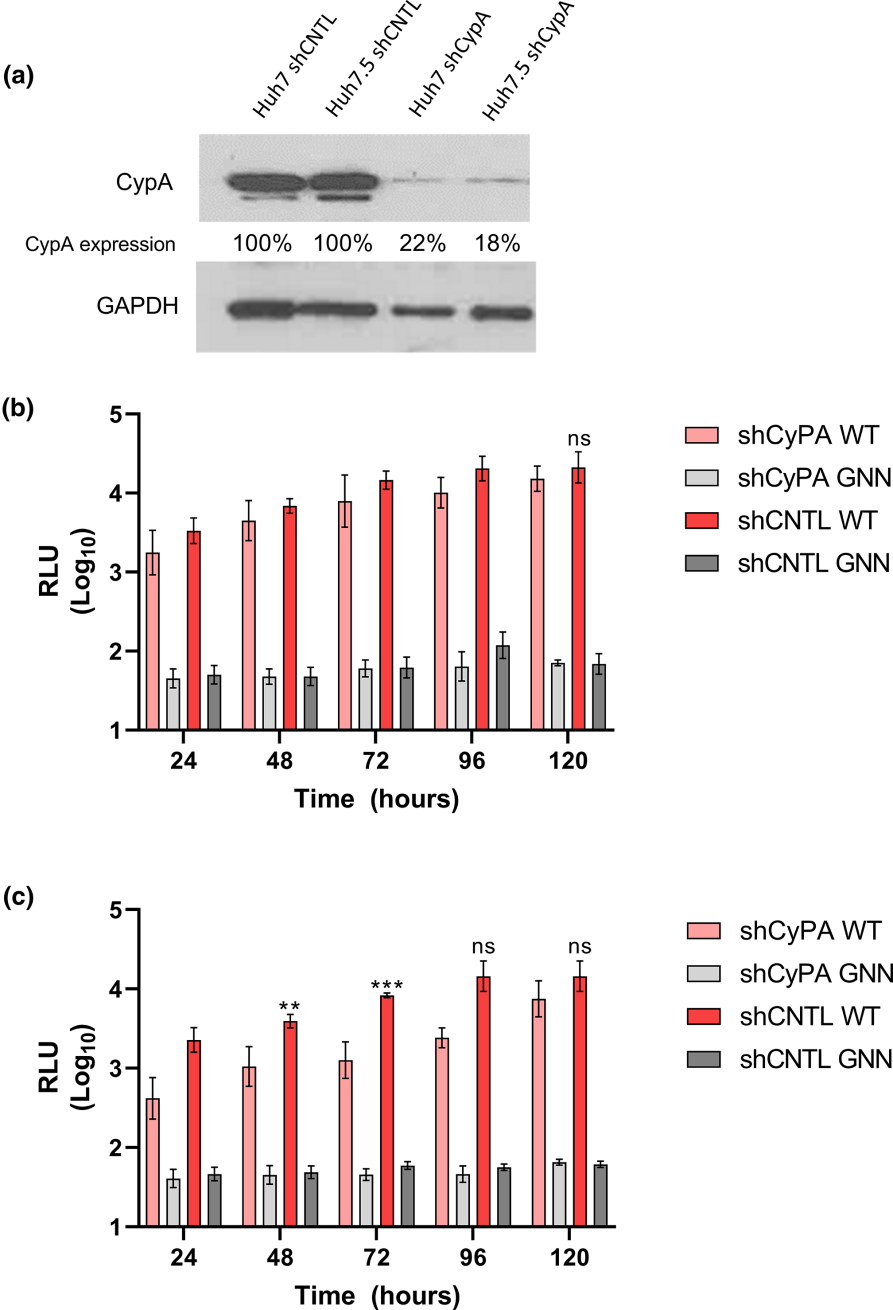
CypA is not essential for HEV-1 replication in Huh7 or Huh7.5 cells. (**a**) Detection of CypA expression by Western blot in Huh7 and Huh7.5 silenced cell lines (shCypA) and scramble controls (shCNTL) with GAPDH used as a loading control, and efficiency of Huh7 shCypA silencing was calculated (78 %) and of Huh7.5 shCypA (82 %). Stable clones of (**b**) Huh7 or (**c**) Huh7.5 cells silenced for CypA by shRNA (shCypA) or a scramble shRNA control (shCNTL) were electroporated with the WT SK-E2-nLuc or non-replicating SK-E2-nLuc-GNN control (GNN) SGR RNAs. Cells were harvested at 24 h intervals for 120 h and luciferase activity was determined. Data are presented as mean luciferase activity as RLU (*n*=3, ±sem, ***P<0.001, **P<0.01, ns *P*>0.05; shCNTL WT compared to shCypA WT).

Pharmacological inhibition of Cyps by CsA only suppressed HEV genome replication at cytotoxic concentrations. To distinguish isomerase activity from other cellular functions that could be involved in HEV replication, we adopted a genetic approach to silence CypA expression by lentiviral delivery of shRNA in both Huh7 and Huh7.5 cells. We first confirmed silencing of CypA expression by Western blot, alongside scramble shRNA controls (Fig. 2a). Quantification confirmed that CypA expression was reduced by ~80 % in both Huh7 and Huh7.5 compared to the scramble control.

**Fig. 3.**
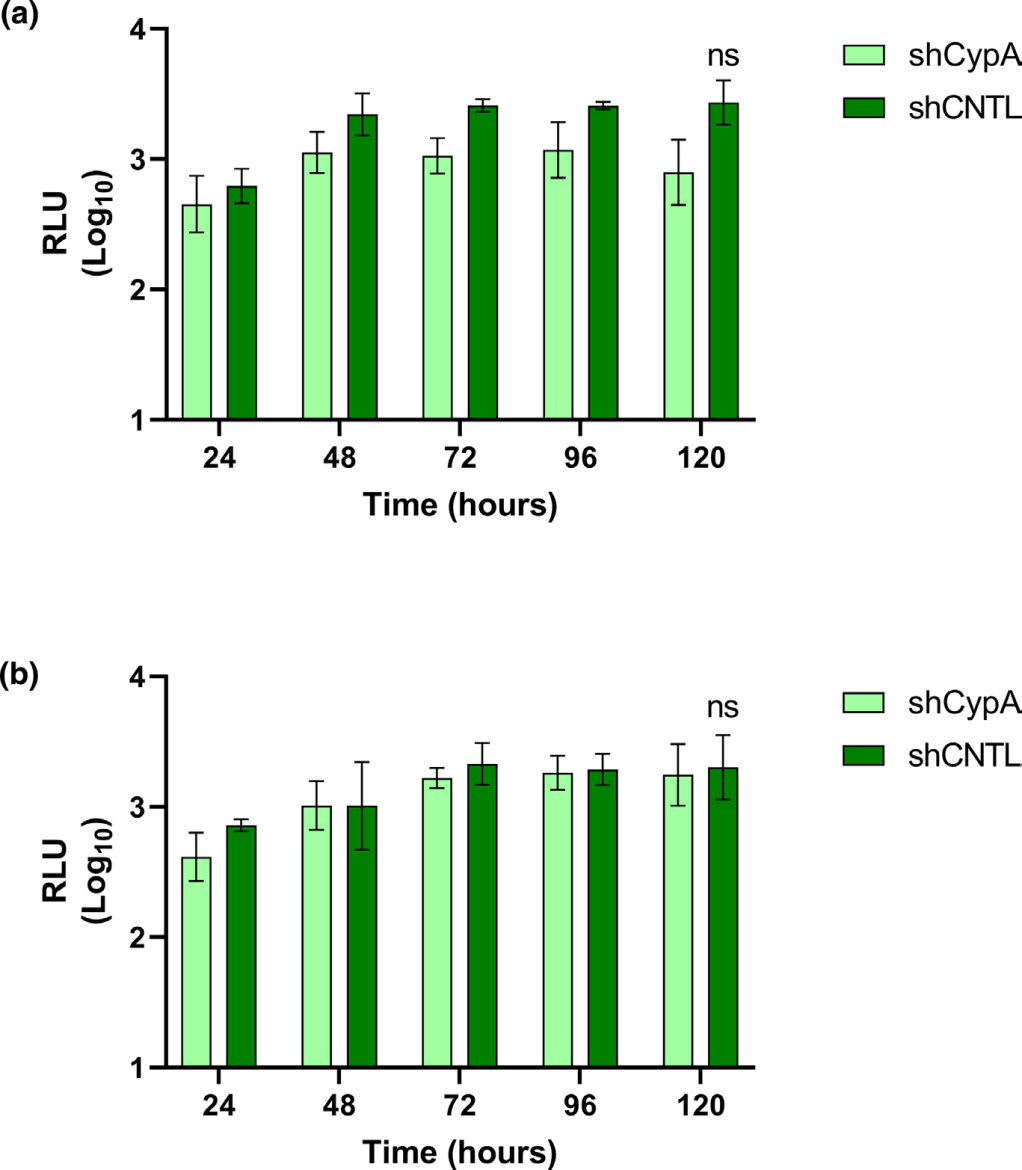
CypA is not essential for HEV-3 replication in Huh7 or Huh7.5 cells. Stable clones of (**a**) Huh7 or (**b**) Huh7.5 cells silenced for CypA by shRNA (shCypA) or a scramble shRNA control (shCNTL) were electroporated with WT 83-2-nLuc replicon RNA. Cells were harvested at 24 h intervals for 120 h and luciferase activity was determined. Data are presented as mean luciferase activity as RLU (*n*=3, ±sem, ns *P*>0.05; shCypA compared to shCNTL).

The CypA silenced cell lines and scrambled controls were transfected with HEV-1 WT SK-E2-nLuc or SK-E2-nLuc-GNN control and nLuc activity was measured over 120 h post-transfection (Fig. 2b, c). To investigate any genotype-specific differences we also repeated these experiments with the HEV-3 WT 83-2-nLuc SGR RNA and nLuc activity measured over 120 h post-electroporation (Fig. 3a, b).

For the WT SK-E2-nLuc SGR, RNA silencing of CypA in Huh7 cells did not significantly reduce replication with nLuc expression equivalent to the scrambled control cell l ine throughout the experiments. There was a modest reduction in replication by 120 h post-electroporation which was not significant. In Huh7.5 cells, silencing of CypA led to an ~1.5-fold decrease in nLuc activity 120 h post-electroporation, but this again was not significant. There was no significant difference in nLuc expression between the CypA silenced and scramble control cell lines at any other time points. For both experiments, the GNN replicon only produced background levels of luciferase at all time points in both cell types.

For the WT 83-2-nLuc SGR, RNA silencing of CypA in either Huh7 or Huh7.5 cells reduced replication by ~3-fold at 24 h to 120 h post-electroporation, but this reduction was not statistically significant compared to the scramble control.

### CypB silencing limits HEV replication in Huh7 and Huh7.5 cells

**Fig. 4.**
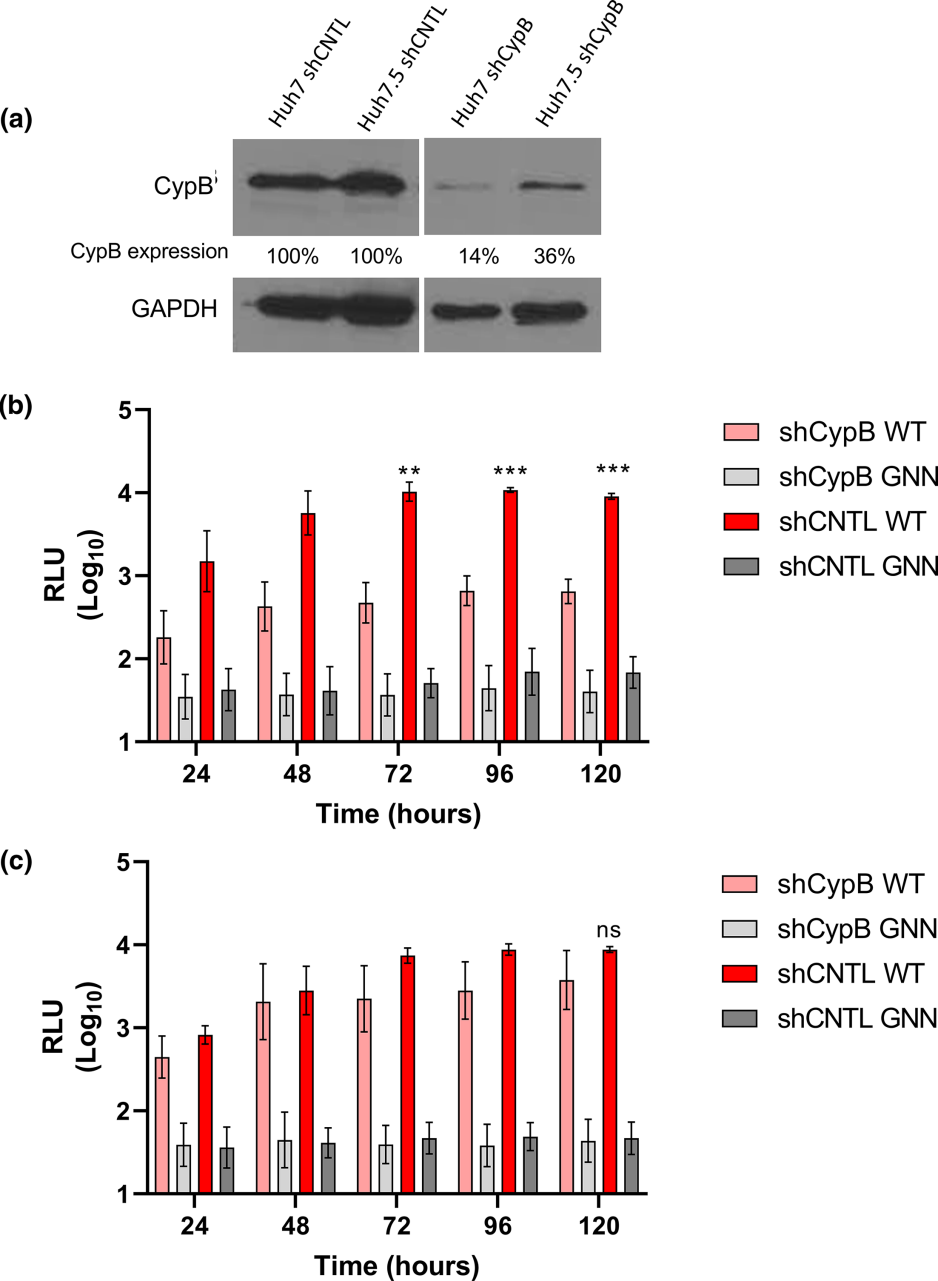
CypB contributed to efficient HEV-1 replication in Huh7 but not Huh7.5 cells. (**a**) Detection of CypB expression by Western blot in Huh7 and Huh7.5 silenced cell lines (shCypB) and scramble controls (shRNA) with GAPDH used as a loading control, efficiency of Huh7 shCypB silencing was calculated (86 %) and of Huh7.5 shCypB (64 %). Stable clones of (**b**) Huh7 or (**c**) Huh7.5 cells silenced for CypB by shRNA (shCypB) or a scramble shRNA control (shCNTL) were electroporated with the WT SK-E2-nLuc or SK-E2-nLuc-GNN replication defective control (GNN) RNA. Cells were harvested at 24 h intervals for 120 h and luciferase activity was determined. Data are presented as mean luciferase activity as RLU (*n*=3, ±sem, ***P<0.001, **P<0.01, ns *P*>0.05; shCNTL WT compared to shCypB WT).

CypB has been found to be important for replication in other RNA viruses such as HCV and Japanese encephalitis virus [37, 38]. We therefore turned our attention to CypB. As described for CypA, we generated stable cell lines in which CypB expression was silenced by shRNA. Silencing of CypB was verified by Western blot (Fig. 4a). Scrambled shRNA sequence was also maintained as a control for both cell types.

**Fig. 5.**
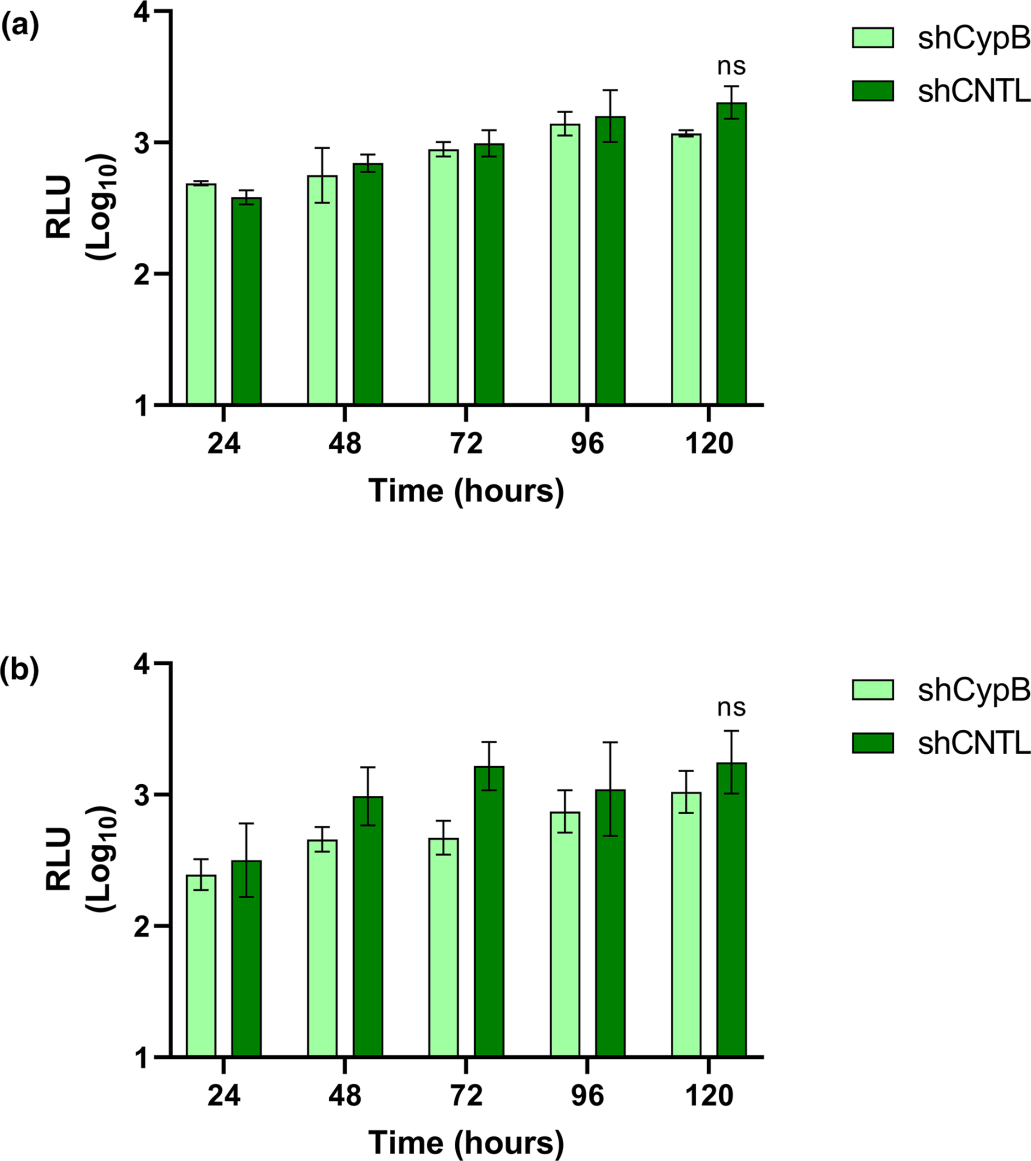
CypB is not essential for G3 HEV-3 replication in Huh7 or Huh7.5 cells. Stable clones of (**a**) Huh7 or (**b**) Huh7.5 cells silenced for CypB by shRNA (shCypB) or a scramble shRNA control (shCNTL) were electroporated with WT 83-2-nLuc replicon RNA. Cells were harvested at 24 h intervals for 120 h and luciferase activity was determined. Data are presented as mean luciferase activity as RLU (*n*=3, ±sem, ns *P*>0.05; shCNTL compared to shCypB).

CypB silenced and control cells (shCNTL) were transfected with the HEV-1 WT SK-E2-nLuc SGR RNA or replication defective SK-E2-nLuc-GNN control and nLuc activity as measured over 120 h post-electroporation as before (Fig. 4b, c). To investigate any genotype-specific differences we also repeated these experiments with the HEV-3 WT 83-2-nLuc SGR RNA as before (Fig. 5a, b).

In contrast to CypA silencing (Fig. 2), CypB silencing in Huh7 cells significantly reduced replication of the WT SK-E2-nLuc SGR between 72 h to 120 h post-electroporation, with luciferase activity approximately ~12-fold lower compared to the scramble control. However, we did not observe the same effect in Huh7.5 cells as there was no significant difference in HEV replication in Huh7.5 shCypB cells compared to shCNTL. As before, the GNN replicon only produced background levels of luciferase at all time points in all cell types. As observed with CypA silencing, silencing of CypB in Huh7 or Huh7.5 cells reduced WT 83-2-nLuc SGR replication by 2- to 4-fold at 72 h to 120 h post-electroporation, but this was reduction was not statistically significant compared to the scramble control.

Taken together with the results from CsA treatment and CypA silencing, we conclude that CypA and CypB are not essential for efficient replication of these HEV SGRs in these cells.

### An insertion in the pORF1 HVR region increases replication and Cyp sensitivity

**Fig. 6.**
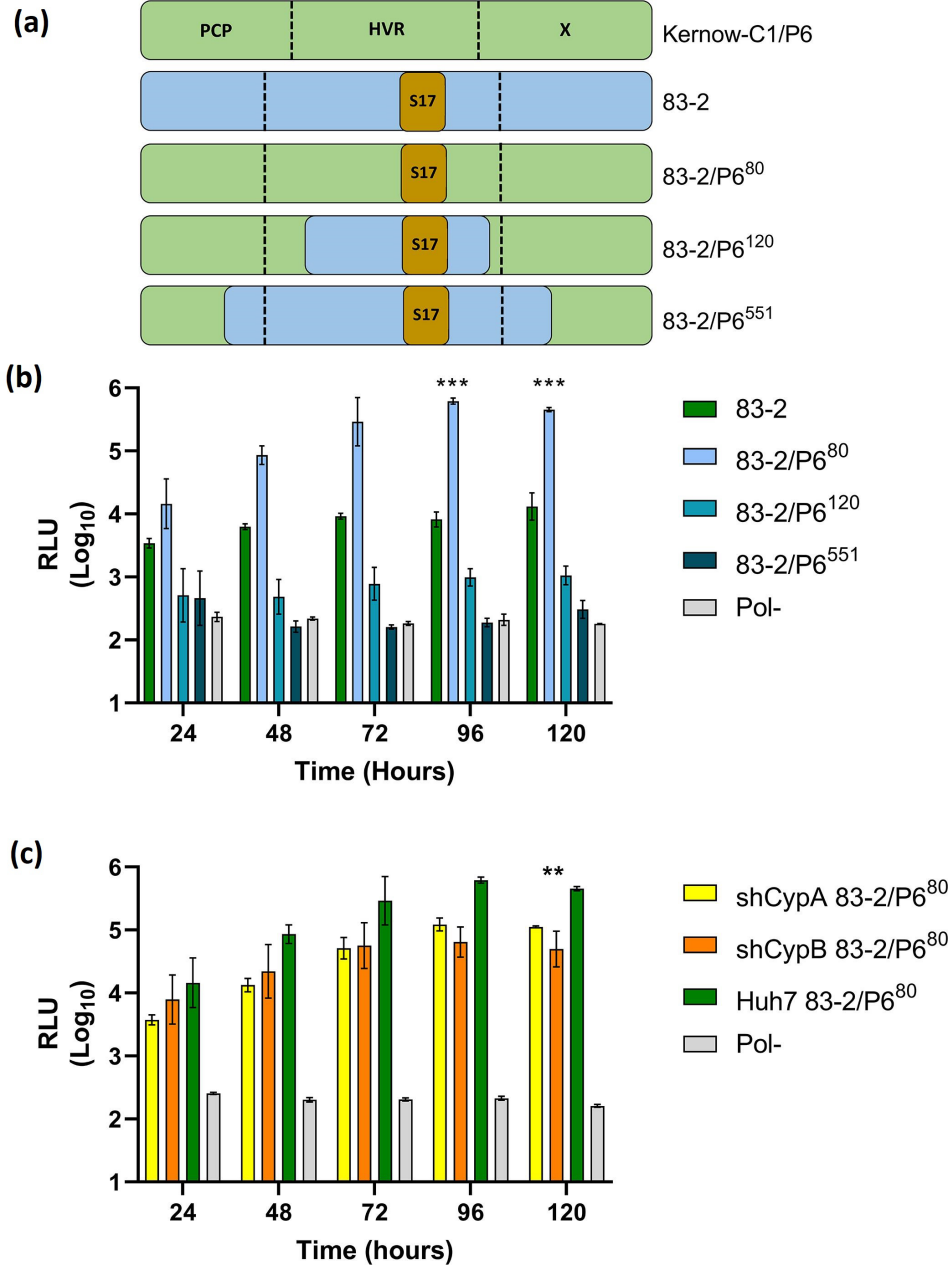
S17 insertion from Kernow-C1/P6 increases SGR replication *in vitro* and confers limited cyclophilin sensitivity. (a) Schematic of HVR domains of HEV83-2-27 and Kernow-C1/P6. Including chimeras of 83-2, (83-2/P6^80^), (83-2/P6^120^) and (83-2/P6^551^). (b) Huh7 cells were electroporated with chimeric 83-2/P6^80^, 83-2/P6^120^ and 83-2/P6^551^ SGR RNAs as well as WT 83-2-nLuc and 83-2-nLuc-Pol- (Pol-) control RNAs. Cells were harvested at 24 h intervals for 120 h and luciferase activity was determined. Data are represented as mean luciferase activity as RLU (*n*=3, ±sem, ***P<0.001, 83-2 compared to 83-2/P6^80^). (c) Huh7 cells silenced for CypA or CypB (shCypA and shCypB, respectively) or scramble control were electroporated with WT chimeric 83-2/P6^80^ chimeric SGR RNA or 83-2-nLuc-Pol- (Pol-) replication-defective control. Cells were harvested at 24 h intervals for 120 h and luciferase activity was determined. Data are presented as mean luciferase activity as RLU (*n*=3, ±sem, **P<0.05; Huh7 83-2/P6^80^ compared to shCypA 83- 2/P6^80^ and shCypB 83-2/P6^80^).

In contrast to our results, a previous study [24] showed that CypA isomerase activity inhibited genome replication of the cell culture adapted HEV-3 strain Kernow-C1/P6 but had no effect on primary isolates of HEV-1 to HEV-4. Consistent with this, CsA treatment also enhanced Kernow-C1/P6 replication [24, 25]. Compared to the primary isolate (Kernow-C1/P1) the Kernow-C1/P6 culture adapted sequence contains an insertion of a fragment of the human ribosomal S17 protein gene within the HVR that enhances viral replication *in vitro* [39]. We therefore considered that the S17 insertion might explain why the Kernow-C1/P6 HEV-3 was sensitive to CypA compared to the SGR used in our study (83-2-nLuc). To investigate this hypothesis, we generated three chimeric HEV-3 SGR clones between 83-2-nLuc and the Kernow-C1/P6 sequence. The first (83-2/P6^80^) replaced 18 residues from the 83-2-nLuc HVR with the equivalent 80 residues from Kernow-C1/P6 which contained the S17 insertion. The second (83-2/P6^120^) replaced 58 residues from the 83-2-nLuc HVR with the equivalent 120 residues from Kernow-C1/P6 (i.e. 83-2/P6^80^ plus an additional N-terminal 35 residues and C-terminal five residues). The third (83-2/P6^551^) replaced the entire HVR plus portions of the PCP and X domains, with the equivalent 551 residues from Kernow-C1/P6. These latter two chimeric SGRs were generated to see if the S17 insertion within the HVR functioned alone or in the context of the surrounding amino acid sequence (Fig. 6a). Huh7 cells were electroporated with the three chimeric HEV-3 SGR as well as the WT 83-2-nLuc and replication defective 832-nLuc-Pol- control SGRs and luciferase activity was measured over 120 h post-electroporation (Fig. 6b).

Compared to the WT 83-2-nLuc SGR, the 83–2/P6^80^ chimera significantly increased replication ~71- and ~26-fold at 96 and 120 h post-electroporation, respectively. In contrast, the 83-2/P6^120^ chimera resulted in an ~2-fold non-significant decrease in replication compared to the WT control at 120 h post-electroporation. Furthermore, the 83-2/P6^551^ chimeric SGR was unable to replicate, with nLuc activity equivalent to the 83-2-nLuc-Pol- replication defective control throughout the time course. These data suggest that the S17 insertion can significant increase replication of a chimeric HEV-3 SGR.

Subsequently, to investigate whether the S17 insertion was sufficient to confer sensitivity to Cyp silencing, we evaluated the replication of the 83-2/P6^80^ chimeric SGR in shCypA and shCypB Huh7 cell lines or Huh7 control cells. These cells were electroporated with the 83-2/P6^80^ chimeric SGR as well as WT 83-2-nLuc and replication defective 83-2-nLuc-Pol- control SGRs and nLuc activity was measured over 120 h post-electroporation (Fig. 6c).

In comparison to the control, Huh7 silencing of either CypA or CypB in Huh7 cells reduced replication of the 83-2/P6^80^ chimeric SGR ~10-fold compared to the Huh7 control cells. This reduction in replication was significantly greater than we previously observed with the WT 82-2-nLuc SGR (Figs 3 and 5), suggesting insertion of the S17 sequence alone was able to confer sensitivity to Cyp silencing.

## Discussion

The role of host cell factors in viral propagation is an important aspect of infection. The Cyps have been identified as such factors, as they can be co-opted by viruses to aid in the completion of their replication cycles and formation of viral particles [21, 22]. HCV, a well-studied hepatotropic virus, relies on the CypA complex in order to evade PKR-mediated innate immune signalling during hepatocyte infection, favouring the formation of membrane-bound replication sites (the membranous web). The current model in the literature suggests that NS5A complexes with both CypA and PKR, preventing CypA-PKR downstream antiviral actions [21]. Additionally, CypB also complexes with the NS5B polymerase and contributes to genome replication [40]. Indeed, in a parallel study to that described in this paper, we have recently demonstrated that HCV genome replication is highly dependent on both CypA and CypB in a genotype- and cell-type-specific manner [41]. The tissue tropism of HEV is very similar to that of HCV, hepatocytes being the primary replication site. Despite this, the importance of Cyp in HEV replication remains disputed [24, 25].

Wu *et al*. [24] adopted the use of induced pluripotent stem cell-hepatocyte like cells (iPSC-HLCs) to investigate cell culture and non-cell culture adapted strains of HEV. iPSC-HLCs infected with non-cell culture adapted HEV genotypes 1–4 were treated with CsA. Total RNA was measured as an indication of replication, and CsA did not have an effect on replication of primary HEV isolates across several genotypes. However, they also found that the culture adapted HEV-3 isolate Kernow-C1/P6 showed enhanced HEV replication under CsA treatment [24]. Likewise, Wang *et al*. [25] found that CsA enhanced replication of the HEV Kernow-C1/P6 isolate in Huh7 cells in a dose-dependent fashion. The differences between adapted and non-cell culture adapted strains of HEV informed our experimental design. Thus, we chose two standard human hepatocellular carcinoma cell lines widely used in both HCV and HEV research and two different HEV genotypes to bring consistency into the investigation of the role of cyclophilin in HEV replication. This included the HEV-1 Sar55 isolate which was previously used by Wu *et al*. In agreement with these studies we find that (in contrast to HCV) CsA did not enhance or impede replication of a HEV-1 or primary HEV-3 SGR, and silencing of either CypA or CypB in Huh7 or Huh7.5 cells showed that neither was essential for HEV replication. In contrast, however, we found that silencing of CypB in particular could decrease the replicative fitness of an HEV-1 replicon in some cell types.

In order to investigate further the enhanced replication and Cyp sensitivity observed in Kernow-C1/P6, we generated chimera SGR constructs. Interestingly we were able to observe significantly increased replication when the parental 83-2-nLuc SGR contained the S17 insertion from Kernow-C1/P6. This suggests that the S17 insertion has the capability to significantly increase HEV replication *in vitro*. However, our additional chimeras that had larger substitutions showed reduced replication compared to the parental 83-2-nLuc sequence, suggesting other amino acids with the HVR are essential for replication efficiency. Further work should be conducted to establish how sequence variation in the HVR increases replication *in vitro*. Irrespective of the mechanism by which HVR sequence variation increases replication, our data suggest the composition of the HVR can confer sensitivity to Cyp inhibition. The observations that the HVR is important for dictating Cyp sensitivity is broadly in agreement with the previous study by Wu *et al*. [24] and Wang *et al*. [25]. However, whereas they found that Cyp inhibition could increase replication of a culture adapted sequence, we found that silencing CypA or CypB moderately decreases replication. The contrasting data reported in this study compared to the studies by Wu *et al*. [24] and Wang *et al*. [25], suggest subtle genotype- and sequence-specific responses to Cyps. Despite these subtly different responses all the data strongly suggest Cyp proteins are not essential for HEV replication.

Several RNA viruses require functional CypA in order to complete their replication cycle. However, this requirement is not universal as CypA is dispensable for Chikungunya virus RNAreplication [42]. Additionally, replication of hepatitis A virus (HAV) in Huh7 cells has been reported to be independent of CypA [43]. These observations were validated pharmacologically and genetically, like our data here. Potentially similar to these viruses we speculate that HEV is able to counter innate immune responses within hepatocytes via alternative pathways that do not rely on CypA. Since the cyclophilins operate primarily as prolyl isomerases, it is possible that this function in HEV is served via other cellular cyclophilins such as cyclophilin D (CypD). Interestingly, HAV has been demonstrated to localize to the mitochondria during hepatocyte cell culture infection, suggesting there could be a link between a lack of CypA dependence and mitochondrial localization, which is also the site of CypD localization [43]. Further investigation is required to understand the mechanisms by which HEV suppresses the innate immune response and prevent elimination by these responses at work within hepatocytes.

## Supplementary Data

Supplementary material 1
