# Supplementary material for: The hepatitis E virus ORF1 hypervariable region confers partial cyclophilin dependency
Source: J Gen Virol. 2023 Nov 9;104(11):001919. doi: 10.1099/jgv.0.001919 (PMC10768694; doi:10.1099/jgv.0.001919)
Supplement: Supplementary material 1 [file jgv-104-1919-s002.pdf]

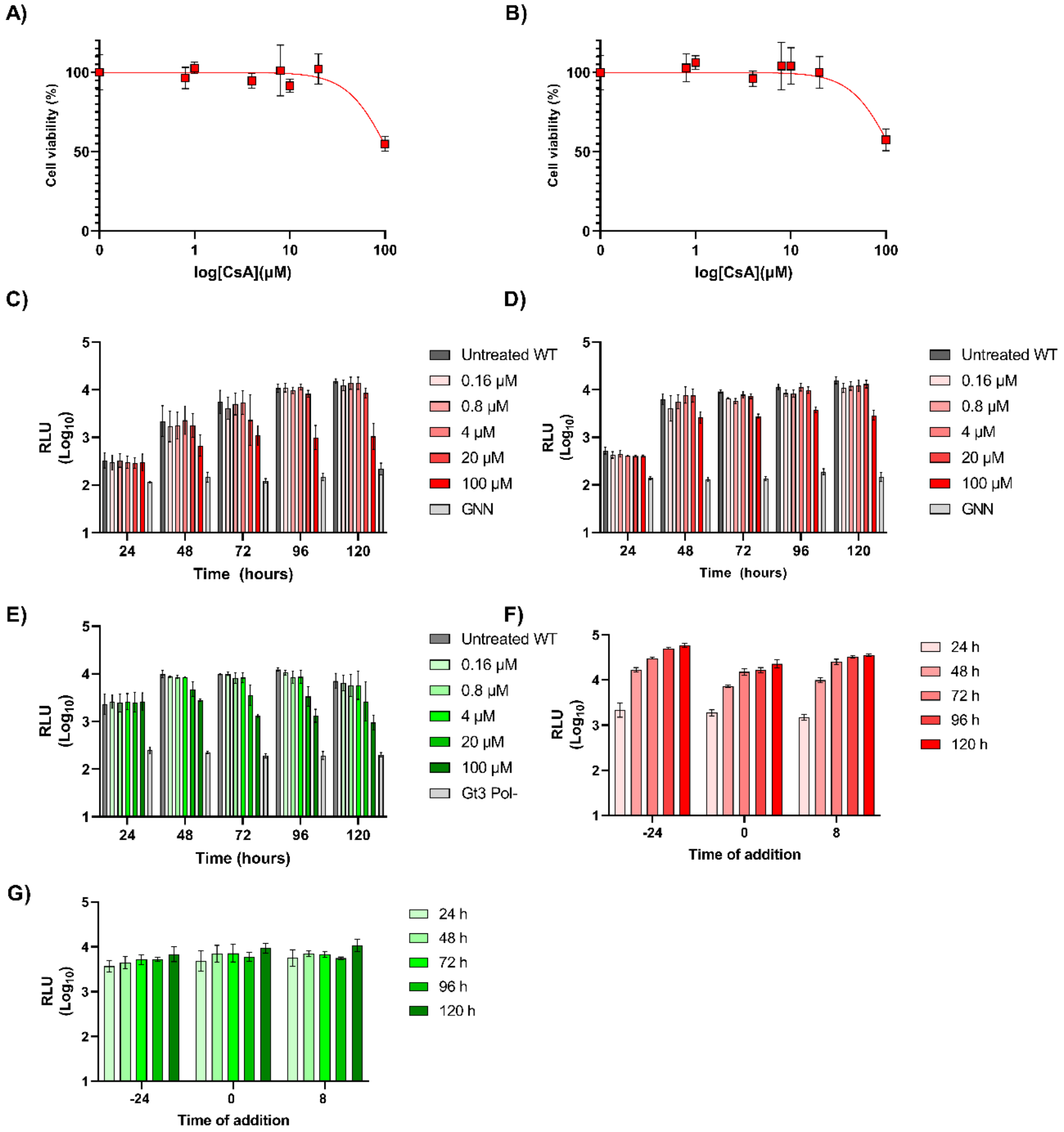

Supplementary Figure S1. Non-cytotoxic concentrations of CsA does not inhibit HEV replication. (A) Huh7 or **(B)** Huh7.5 were seeded into 96-well plates, allowed to adhere for 24 h before replicate wells were treated with a serial dilution of cyclosporine (0 - 100 μM). Replicate wells were left untreated or treated with DMSO solvent only as controls. 72 h after treatment cell viability of Huh7 cells was calculated by MTS assay. Data presented as mean percentage cell viability, normalised to untreated controls (n = 3 +/- SEM). **(C)** Huh7 or **(D)** Huh7.5 cells were electroporated with wild-type (WT) SK-E2-nLuc or SK-E2-nLuc-GNN (GNN) SGR RNA prior to addition of CsA at varying concentrations (0 - 100 μM) 24 h post-

electroporation. Cells were harvested at 24 h intervals for 120 h and luciferase activity determined. Data are presented as mean luciferase activity as relative light units (RLU) (n= 3 +/- SEM). **(E)** Huh7 or cells were electroporated with WT 83-2-nLuc or 83-2-nLuc-Pol- (Pol-) prior to addition of CsA at varying concentrations (0 - 100 µM) 24 h post-electroporation. Cells were harvested at 24 h intervals for 120 h and luciferase activity determined. Data are presented as mean luciferase activity as relative light units (RLU) (n= 3 +/- SEM). Huh7 cells were treated with 20 µM CsA 24 h pre-electroporation, immediately following electroporation or 8 h post-electroporation with either **(F)** WT SK-E2-nLuc or **(G)** WT 83-2-nLuc SGR RNA. Cells were harvested at 24 h intervals for 120 h and luciferase activity determined. Data are presented as mean luciferase activity as relative light units (RLU) (n= 3 +/- SEM).
